# Supplementary material for: RedquorinXS Mutants with Enhanced Calcium Sensitivity and Bioluminescence Output Efficiently Report Cellular and Neuronal Network Activities
Source: Int J Mol Sci. 2020 Oct 22;21(21):7846. doi: 10.3390/ijms21217846 (PMC7660078; doi:10.3390/ijms21217846)
Supplement: Supplementary file 1 [file ijms-21-07846-s001.zip › Supplementary.pdf]

Supplementary material to the article:

"RedquorinXS Mutants with Enhanced Calcium Sensitivity and Bioluminescence Output Efficiently Report Cellular and Neuronal Network Activities", by Bakayan et al.

Supplementary Table S1 – summarizes properties of aequorin mutant proteins with altered calcium sensitivity

Group	Mutation	Calcium sensitivity, *EC50* (nM) [a]	Relative intensity [a]		Decay kinetics, $t_{1/2}$ (ms) [b]	Spectral emission peak (nm)
			at pCa 6.5	at pCa 7.2		
	**Aeq-wt**	659 ± *23*	1.0	1.0	906 ± *53*	476
Single mutants with unchanged relative intensity and/or affinity	**D8A**	493 ± *50*	1.05	0.64	*ND*	*ND*
	**D8Q**	681 ± *45*	0.91	1.26	*ND*	*ND*
	**G14K**	567 ± *33*	0.79	0.90	*ND*	*ND*
	**G14N**	539 ± *65*	0.95	0.90	*ND*	*ND*
	**V25A**	484 ± *35*	0.61	0.67	*850 ± 63*	476
	**H27N**	514 ± *81*	0.66	0.63	*ND*	*ND*
	**H27G**	480 ± *59*	0.83	0.66	930 ± *51*	473
	**I43D**	774 ± *65*	0.63	1.26	*ND*	*ND*
	**G49E**	565 ± *42*	0.51	0.65	*ND*	*ND*
	**G49N**	502 ± *75*	0.63	0.63	890 ± *65*	472
	**K87E**	681 ± *46*	1.15	0.76	*ND*	*ND*
	**D92H**	606 ± *32*	1.26	1.15	*ND*	*ND*
	**D92S**	756 ± *23*	1.07	0.87	*ND*	*ND*
	**Q120A**	509 ± *42*	0.64	0.62	*ND*	*ND*
	**Q120E**	606 ± *68*	1.20	1.05	*ND*	*ND*
	**N121S**	638 ± *65*	1.51	1.52	*ND*	*ND*
	**A123T**	639 ± *51*	1.37	1.39	*ND*	*ND*
	**A123S**	524 ± *32*	1.02	0.87	*ND*	*ND*
	**A136S**	676 ± *89*	1.17	1.02	*ND*	*ND*
	**Q140P**	590 ± *75*	0.93	0.74	*ND*	*ND*
	**R150E**	504 ± *66*	0.87	0.76	*ND*	*ND*
	**Q159K**	550 ± *36*	1.58	1.63	*ND*	*ND*
	**A179V**	758 ± *58*	1.53	1.52	*ND*	*ND*
	**A179E**	526 ± *49*	0.91	0.66	*920 ± 45*	473
Single mutants with lower relative intensity	**V25I**	742 ± *45*	0.43	0.71	850 ± *23*	472
	**Q120R**	935 ± *23*	0.21	0.55	838 ± *57*	476
	**Q140R**	*1 700 ± 59*	0.08	0.25	912 ± *71*	474
	**S157A**	1210 ± *33*	0.07	0.28	890 ± *39*	474
	**S157T**	1022 ± *66*	0.38	0.96	910 ± *28*	474
	**S157K**	1170 ± *52*	0.27	0.71	925 ± *36*	476

Aequorin mutant proteins were reconstituted with CLZ-f. The table was divided into two groups and displays properties of aequorin mutants (with single mutations) with unchanged or lower relative intensity and/or affinity. For more details on definitions and calculations, refer to the legends of Table 1.

Supplementary Table S2. Summary of thermostability data obtained at two temperatures for photoproteins and their mutants

	wt-Aeq	N121D	A123D	Q159D	Q159T	Q159G	Q159E
30°C	1.00	0.81	0.82	0.95	0.92	0.96	0.89
40°C	1.00	0.82	0.84	1.00	0.96	0.92	0.94
	**S157D**	**A179T**	**QD+ND**	**QD+AD**	**QD+AT**	**CitA**	**GA**
30°C	0.77	0.96	0.81	0.97	1.05	0.96	0.95
40°C	0.71	1.02	0.81	0.94	1.07	1.00	0.97
	**Redq**	**Redq/QD**	**Redq/QD+AT**	**Redq/QD+AD**	**Redq/QT**	**Redq/QT+AD**	**Redq/QT+AT**
30°C	0.97	0.91	1.00	0.97	0.90	0.94	0.96
40°C	0.97	0.95	1.03	0.99	0.92	1.00	1.03

The values represent the degree of change in luminescence activity compared to wt-aequorin, at each temperature. All values are relative to wt-aequorin (at each temperature) and were calculated by dividing the value obtained for each mutant/variant by the value obtained for wt-aeq (as reference). The data were taken from Figure 4.

Supplementary Movie:

This 5 s movie corresponds to 120 s of the prominent bioluminescence peak visible on the recording of the mouse neocortical slice expressing RedquorinXS-Q159T shown in Figure 7C, lower panels.
